# A New Coated Nitinol Occluder for Transcatheter Closure of Ventricular Septal Defects in a Canine Model

**DOI:** 10.1155/2013/507919

**Published:** 2013-08-27

**Authors:** Yong Zhou, Feng Chen, Xinmiao Huang, Xianxian Zhao, Hong Wu, Yuan Bai, Yongwen Qin

**Affiliations:** ^1^Department of Cardiology, The No. 411 Hospital of People's Liberation Army, Shanghai 200081, China; ^2^Department of Cardiology, Changhai Hospital, Second Military Medical University, Shanghai 200433, China

## Abstract

*Aims*. This study evaluated feasibility and safety of implanting the polyester-coated nitinol ventricular septal defect occluder (pcVSDO) in the canine model. *Methods and Results*. VSD models were successfully established by transseptal ventricular septal puncture via the right jugular vein in 15 out of 18 canines. Two types of VSDOs were implanted, either with pcVSDOs (*n* = 8) as the new type occluder group or with the commercial ventricular septal defect occluders (VSDOs, *n* = 7, Shanghai Sharp Memory Alloy Co. Ltd.) as the control group. Sheath size was 10 French (10 Fr) in two groups. Then the general state of the canines was observed after implantation. ECG and TTE were performed, respectively, at 7, 30, 90 days of follow-up. The canines were sacrificed at these time points for pathological and scanning electron microscopy examination. The devices were successfully implanted in all 15 canines and were retrievable and repositionable. There was no thrombus formation on the device or occurrence of complete heart block. The pcVSDO surface implanted at day 7 was already covered with neotissue by gross examination, and it completed endothelialization at day 30, while the commercial VSDO was covered with the neotissue in 30th day and the complete endothelialization in 90th day. *Conclusion*. The study shows that pcVSDO is feasible and safe to close canine VSD model and has good biocompatibility and shorter time of endothelialization.

## 1. Introduction

Over the past ten years, transcatheter closure of VSD has been widely used in China [1–3]. However, the most serious and unpredictable complication is still postprocedural atrioventricular block (AVB). With early clinical experience of Amplatzer VSDO, AVB occurred at a rate of up to 3–8% with 3.8% requiring the placement of permanent pacemaker [4–6]. Although the postprocedural AVB incident rate is lower when using our domestic compared with using Amplatzer VSDO, we still cannot avoid its occurrence [1–3]. We believe the root cause could be the occluder itself. 

At present, the nitinol occlude is still woven by bare nitinol wire. This may cause three issues: (1) the hard nitinol wire and local tissues have direct contact, extrusion, and friction, which may result in the myocardium and conducting system damage, reducing postprocedural AVB [4–6]; (2) the Dacron fibers are sutured in VSDO, resulting in high speed flow of red cells through the slit and hit the right ventricle side of nickel wire, then occurring hemolysis [7, 8]; (3) the alloy surface is very smooth, not easy to endothelium; thus it increases the risk of thrombosis on occluder surface at early stage [9, 10].

After reviewing the defects of existing occluder, we produced the pcVSDO. In order to reduce the incidence of atrioventricular conduction system injury, embolism, and other complications, we coated surface of the VSDO with polyester clothing, which can prevent hard alloy wire from direct contact with soft myocardial tissue. To estimate the feasibility, safety, and effectiveness and provide experimental basis for clinical application, we used the animals implanted to evaluate the performance of this new type of ventricular septal occluder, while using the commercial occluder as control group. 

## 2. Methods

### 2.1. The PcVSDO

**Figure 1:**
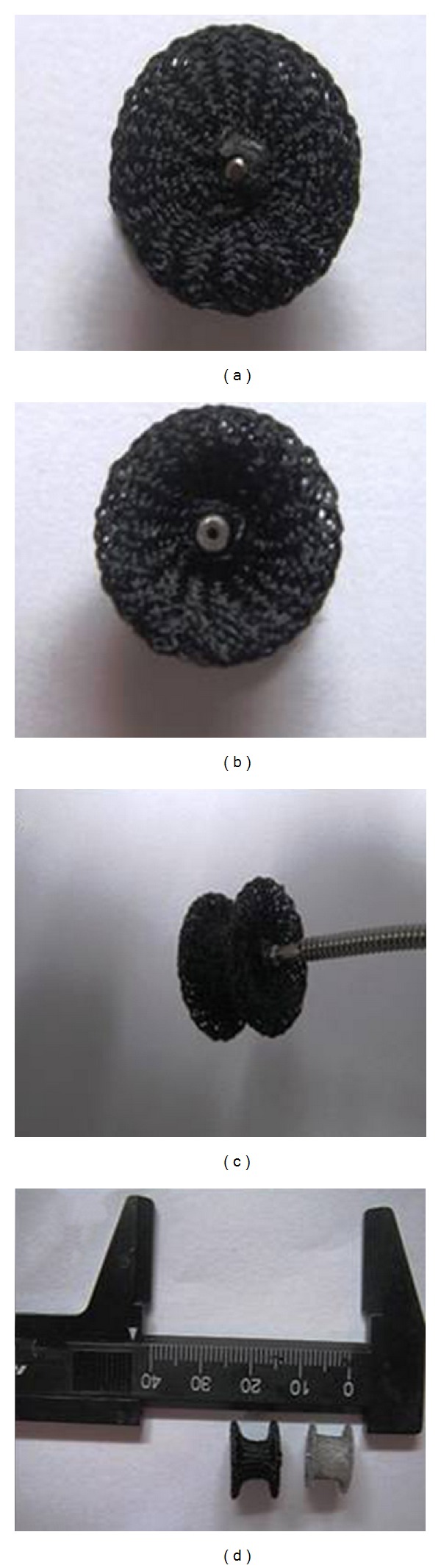
(a) through (d) show the profile of pcVSDO: waist length and diameter 7 mm and 8 mm, right and left rim 12.5 mm. The pcVSDO is wearing polyester clothes of the commercial occluder.

The occluder is made from Shanghai the Shape Memory Alloy Company (SHSMA Company), with waist length, waist diameter, and discs' diameter at 7 mm, 8 mm, and 12 mm, respectively. It is covered with the medical 4-0 polyester thread warp knitted coat, both ends were sutured in the rivet and cut by hot knife to make polyester ingredients solidification and to prevent loose strand (Figure 1). Before animal experiments, we designed some tests to compare the performance of the pcVSDO (pcVSDO group) and commercial VSDOs (commercial occluder group). We concluded that (1) the diameters of pcVSDOs' rims are larger than those of commercial VSDOs by 0.5 mm. pcVSDOs require a minimum of 10 Fr sheath to delivery while the commercial VSD occluder requires 8 Fr sheath. (2) The tension of occluders in these two groups has no statistically significant difference in the deformation process of 1.25, 2.5, 3.75, 5, 6.25, 7.5, 8.75, 10, 11.25, 12.5, 13.75, and 15 mm. (3) The pcVSDOs showed no obvious wear and tear by eyes, and the surfaces not significant difference by stereomicroscope before and after the release-withdrawal 50 times. (4) There were no tiny desquamations after release-withdrawal 50 times in saline by observed lowest centrifugal liquid in both groups by stereomicroscope. No significant difference was observed in various physical parameters in these two groups. So the pcVSDOs can be safe to implant in animals.

### 2.2. Implantation of the VSD Occluders

Total of 15 commercial VSDOs occluders were randomly divided into two groups: the new type occluder group with 8 and the control group with 7. The new type occluder group, VSDOs, was produced into pcVSDOs. Two groups of occluder was implanted in a canine animal model in vivo.

The VSD canine models were established as described in the previous literature [11]. Briefly, the canines were anesthetized with intravenous ketamine (8 mg/kg) and diazepam (0.8 mg/kg). Electrocardiogram (ECG) and blood pressure monitored by femoral artery puncture were performed. If there is restlessness in canine, propofol (1 mg/kg) was further intravenously injected. Femoral vein puncture was performed for catheter access. The devices were implanted during the same procedure of VSD creation, using 10 Fr sheaths through the VSD into the left ventricle, and pcVSDOs and commercial VSDOs are used to close the VSD models. Immediate left ventriculograms were conducted to check the position of the device and to quantify any residual shunt. All procedures performed on canines were approved by the Ethics Committee of Changhai Hospital (Approval Reference No. CH 20110234) and carried out in accordance with the relevant Chinese laws.

### 2.3. Follow-Up after Device Implantation

The canines were fed routinely and administered intramuscular injection of penicillin (800,000 u) daily (twice a day) for 3 days, low molecular heparin 1000 u injection twice a day. The canines were fed with aspirin 3 mg/kg daily in the following time points until they were sacrificed. Observation was done for the dietary intake, the mental state, and the performing situation of the canines, such as with or without blood in the stool, whether the urine color changed, and with or without abnormal behavior or hemiplegia. On days 7 and 30 after intervention, ECGs and TTE checks were repeated. At the time point of days 7, 30, and 90, the canines were anesthetized by the method described above and then sacrificed. The device, together with the surrounding tissues, was explanted, cleaned, and subjected to gross inspection, pathologic and scanning electron microscope examination.

## 3. Results

In a total of 18 canines, 2 appeared pericardial tamponade, 1 had complete atrioventricular block and died during puncture ventricular septal, and 15 cases were successfully modeling, of which 8 implanted with pcVSDOs, 7 with commercial occluders.

The canines in both groups had a good recovery, good spirits, good diet, and activity. No postprocedural paralysis, hematuria, bloody stools were observed. All canines survived to the follow-up to the end.

At one week and one month after intervention, ECGs showed that all of the canines were of sinus rhythm and no arrhythmia. TTE demonstrated that, in all of the occluders, no shift, no residual shunt, and no vegetation appeared in the surface of the occluder or within the heart. There were no obvious tricuspid and aortic regurgitation, either.

### 3.1. Gross Examination

**Figure 2:**
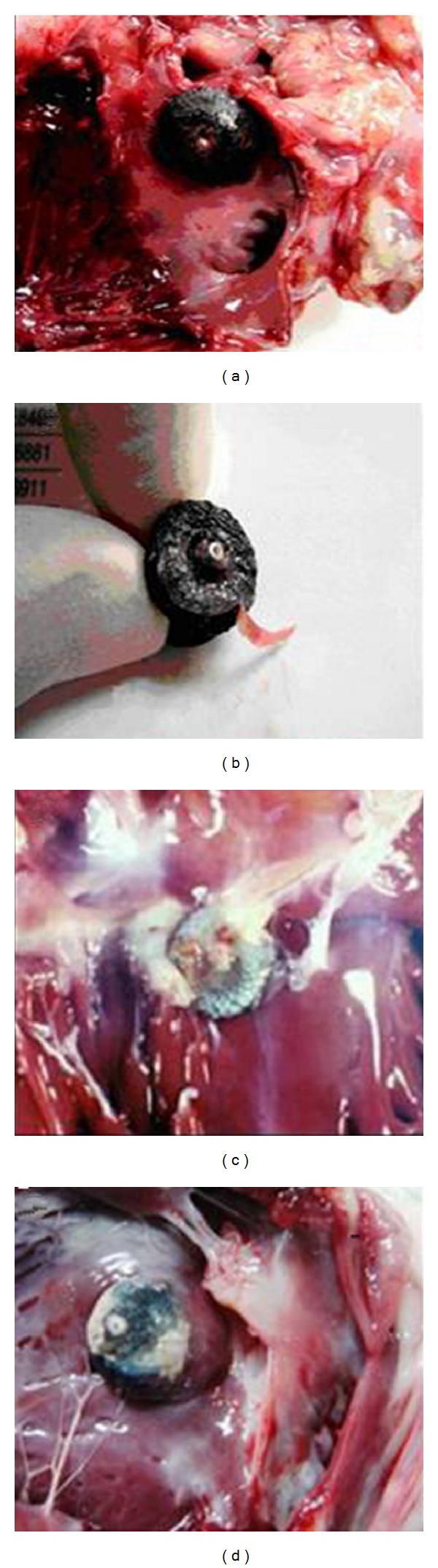
Gross examination of tissues around the device: (a) and (b) show the pcVSDO surface covered a slice of intact thicken membrane at 7 days after closure of defect. No dislocation, thrombus formation, and device fracture were observed. (c) shows the surface and surrounding of the commercial occluder presented a large number of neotissue, while (d) shows the tissue was significantly reduced compared to the commercial occluder.

After one week, one month, and 3 months of the intervention, two canines in each group were sacrificed each time at those time points. The remaining three canines were ready to be breeding for three years. At day 7, the pcVSDOs' surface was covered by grey-white membranes completely (Figure 2), while the commercial VSDOs had no tissue by gross examination. After three months, the new type occluder group occluders implanted had less neotissue around the site, while the commercial VSDO occluder group had more neotissue blocking around.

### 3.2. HE Staining

**Figure 3:**
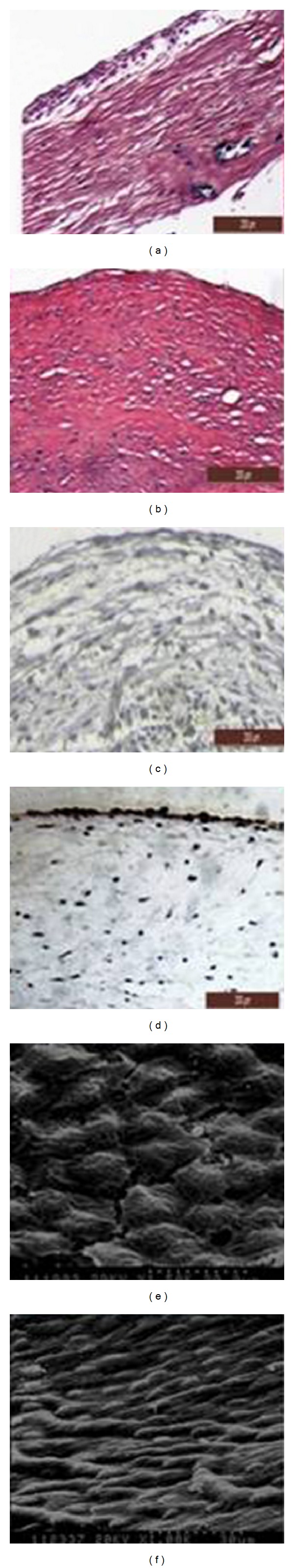
(a), (c), and (e) are the HE and CD34 immunohistochemical staining of the pcVSDO's surface neotissue at 1 month after implantation. These three panels prove the occluder surface has complete endothelialization. (b), (d), and (f) at 3 months show that endothelial cells were arranged more regularly.

**Figure 4:**
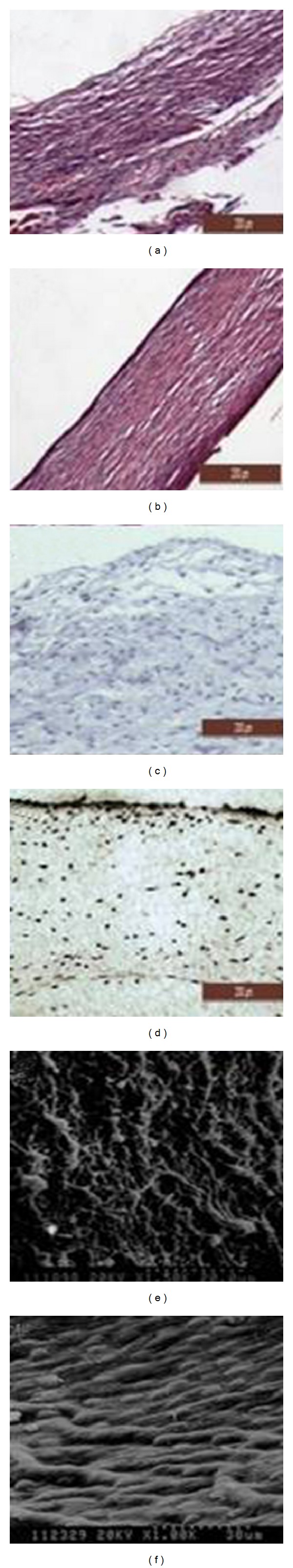
(a), (c), and (e) are the HE and CD34 immunohistochemical staining of the commercial VSDO's surface neotissue at 1 month after implantation. On the occluder's surface neotissue, HE staining found no flat cells. CD34 immunohistochemical staining found no positive staining particles and scanning electron microscopy also no endothelial cells. (b), (d), and (f) at 3 months show that endothelial cells presented at the surface of neotissue.

Two groups of occluder surface neotissues were obtained by using a sharp blade striping, stained with HE. Micrographs showed that the surface of pcVSDO at day 7 had the neotissue covering, and at days 30 and 90 the neotissue surface was covered with flat cells. Commercial occluder surface was covered with neotissue at day 30 and flat cells at day 90 (Figures 3 and 4).

### 3.3. Immunohistochemical Staining

The neotissues of the surface of pcVSDO were stained positively with CD34 at days 30 and 90, while, in neotissues of the surface of commercial occlude, CD34 staining was positive only at day 90 (Figures 3 and 4).

### 3.4. Scanning Electron Microscopy

Scanning electron microscopy was performed in the neotissue of the surface of the occluder. It was found that the surface tissue of the pcVSDO had connective tissue at day 7 and was completely covered by neoendothelial cells at day 30, and the endothelial cells were fusiform and arranged in neat rows at day 90. The surface of commercial occluders was covered with connective tissue at day 30 and completely covered by endothelial cells at day 90 (Figures 3 and 4).

## 4. Discussion

This study uses pcVSDO transcatheter closure of the canine VSD model, three cases of canines' deaths in the process of septal model establishment, which has nothing to do with the implantation of the occluder. 15 of 18 canines were successfully set up for VSD models, which closed with pcVSDOs and commercial occluders. After closure, all canines survived till the end points of follow-up. During the follow-up, the canines were normal, their spirits were in good condition, and their behaviors had no obvious abnormalities. They also had no hemiplegia, no gross hematuria, and their urine color and frequency were normal. Follow-up ECG at days 7 and 30 did not show any arrhythmia. In four-week time, TTE showed clear image of the occluder and no obvious tricuspid and aortic regurgitation. There were no residual shunts, no vegetation in the heart and the surface of the occluder, and no occluder dislocation as well. All of these results suggested that this new occluder is reliable and is safe to use.

Nitinol VSDO was implanted in the VSD site. Two discs of the occluder are folded together while squeezing the septal organization, and this leads to tissue edema. When the heart is beating, the friction between the bare nitinol wire and the surrounding tissue will cause damage to the myocardial and result in the inflammatory response in the early period after the closure device is implanted. Subsequently, around the occluder and tissue binding sites, the increasing of the number of fibroblasts of the new organization will increase the proliferation of neotissue. And the more intense in the inflammatory response at the early stage, the more obvious neotissue is. The neotissue will pull the surrounding normal myocardial tissue, oppress the conduction organization, and lead to the occurrence of arrhythmia [12, 13]. It is found in this study that the neotissue proliferation in the surface of occluder was significantly less than the commercial occluder. We speculate that the reason is due to the effect of the coated occluder. The coating becomes the barrier between the hard nitinol wire and the soft heart; thus this changes the contacting with “hard-soft” mode to “soft-soft” mode, thereby reducing the inflammatory response and the tissue proliferation in the surface and the surrounding of occluder. Therefore it can be predicted that postoperative arrhythmias, especially complete atrioventricular block, will less occur accordingly.

After VSD occluder implanted, many patients have significant residual, and it has been reported up to 50%. The reason is partly because the occluder is too small but largely because the Dacron in the middle of VSDO is sutured between the nickel-titanium alloy wires, which make the edge of the gap left and result in residual shunt. When the high-speed blood flows through the gap, it will impact on the right disk of nitinol, leading to hemolysis. In the event of hemolysis, if not handled in a timely manner, it will result in serious complications such as renal failure. Our design of pcVSDO whose surface is covered with a layer of polyester coat can prevent the red blood cells from making the direct impact of the nitinol wire and to avoid hemolysis [7, 8].

From the observation of the neotissue on the occluder surface with the gross examination, we can find that, on the surface of rough pcVSDO, the time required for the neotissue completely covered and complete endothelialization is much less when compared with smooth commercial VSDO. So we believe that the rough graft surface contacts with blood can make the endothelial cells much easier to adhere and to grow. This finding has not yet been reported in the literature. Will a rough surface directly contacting with the blood lead to an increase in the risk of thrombosis? From the results of our study, eight canines implanted with pmVSD, with intraprocedural heparin anticoagulation, and aspirin antiplatelet after surgery; none of them has thrombosis occurred in 6 months. So we think that, with formal anticoagulation, antiplatelet treatment, pcVSDO will not increase the risk of thrombosis, similar to the rough Dacron patch of VSD.

## 5. Conclusions

The pcVSDO can be implanted by transcatheter methods on canine model for VSD closure, and the procedure method is simple, safe, and reliable. It also has a reliable effect and an excellent biocompatibility. Compared to commercial occluder, it can also make endothelialization happen significantly ahead of schedule.

## 6. Limitations

In retrospect, the study we have carried out has the following limitations. First, the position of the defect is not exactly in the perimembranous area. Second, the histological test is lack of analysis of local inflammatory answer. Third, the canine model could have a different behaviour compared to humans. Last, the canines had been followed up for 3 months in our study since the complete endothelialization was achieved in 3 months. However, longer follow-up periods seem to be necessary to examine the effect of the device on the myocardium.
